# Membrane-associated RING-CH (MARCH) 8 protein inhibits HIV-1 infection

**DOI:** 10.1186/1742-4690-10-S1-P90

**Published:** 2013-09-19

**Authors:** Takayoshi Koyama, Takuya Tada, Hideaki Fujita, Kenzo Tokunaga

**Affiliations:** 1 National Institute of Infectious Diseases, Tokyo, 162-8640, Japan; 2 Nagasaki International University, Nagasaki, 859-3298, Japan

## Background

The MARCH (membrane-associated RING-CH) 8 protein is a member of a recently discovered MARCH family of RING-finger E3 ubiquitin ligases, 11 members of which have been identified. MARCH8 downregulates several transmembrane proteins, such as MHC-II, CD86, TRAIL receptor 1, and CD98. We have recently reported that MARCH8 also negatively regulates cell-surface expression of transferrin receptor [1]. During the preparation of MARCH8-encoding lentiviral vector, we observed that the infectivity of the vector was considerably lower than that of the control vector, suggesting that MARCH8 might have the ability to reduce viral infectivity. Here, we have assessed the impact of MARCH8 expression on HIV-1 replication.

## Materials and methods

Endogenous levels of MARCH8 expression were analyzed for different transformed and primary cell types by real-time RT-PCR. To determine whether HIV-1 could be attenuated by MARCH8, an env-deficient HIV-1 luciferase reporter construct was cotransfected with MARCH8 expression plasmids (wild-type, RING-CH mutant, and C-terminal serial deletion mutants), together with a plasmid expressing VSV-G or HIV-1 Env, into 293T cells. The resulting viruses were used for infection of HeLa-based MAGIC5 cells. An shRNA lentiviral vector targeting MARCH8 was created and used for transduction of HepG2 cells. Viruses prepared from MARCH8-depleted HepG2 cells were used for infection. The infected cells were subjected to luciferase assay to determine the infectivity.

## Results

MARCH8 was generally expressed in all cell lines and primary cells tested, and especially highly expressed in monocyte-derived dendritic cells. Overexpression of MARCH8 in virus-producer cells did not influence the level of virion production, but resulted in the reduction of viral infectivity in a dose-dependent manner by 50-fold or more. MARCH8 overexpression in target cells had no effect on HIV-1 infection. Mutational analyses of MARCH8 revealed that its RING-CH domain and amino acid positions 237-242 are critical for the restriction of HIV-1 replication. The infectivity of the viruses produced from MARCH8-depleted HepG2 cells was greatly enhanced. The exact molecular mechanism by which HIV-1 infection is blocked by MARCH8 is currently under investigation.

## Conclusions

Our findings indicate that MARCH8 is a novel host restriction factor that blocks HIV-1 infection during the early phase of replication.
